# Dopaminergic Co-Regulation of Locomotor Development and Motor Neuron Synaptogenesis is Uncoupled by Hypoxia in Zebrafish

**DOI:** 10.1523/ENEURO.0355-19.2020

**Published:** 2020-02-11

**Authors:** Jong-Hyun Son, Tamara J. Stevenson, Miranda D. Bowles, Erika A. Scholl, Joshua L. Bonkowsky

**Affiliations:** 1 Department of Biology, University of Scranton, Scranton, PA 18510; 2 Department of Pediatrics, University of Utah School of Medicine, Salt Lake City, UT 84132; 3 Brain and Spine Center, Primary Children’s Hospital, Salt Lake City, UT 84108

**Keywords:** axon pathfinding, hypoxia, prematurity, synaptogenesis

## Abstract

Hypoxic injury to the developing human brain is a complication of premature birth and is associated with long-term impairments of motor function. Disruptions of axon and synaptic connectivity have been linked to developmental hypoxia, but the fundamental mechanisms impacting motor function from altered connectivity are poorly understood. We investigated the effects of hypoxia on locomotor development in zebrafish. We found that developmental hypoxia resulted in decreased spontaneous swimming behavior in larva, and that this motor impairment persisted into adulthood. In evaluation of the diencephalic dopaminergic neurons, which regulate early development of locomotion and constitute an evolutionarily conserved component of the vertebrate dopaminergic system, hypoxia caused a decrease in the number of synapses from the descending dopaminergic diencephalospinal tract (DDT) to spinal cord motor neurons. Moreover, dopamine signaling from the DDT was coupled jointly to motor neuron synaptogenesis and to locomotor development. Together, these results demonstrate the developmental processes regulating early locomotor development and a requirement for dopaminergic projections and motor neuron synaptogenesis. Our findings suggest new insights for understanding the mechanisms leading to motor disability from hypoxic injury of prematurity.

## Significance Statement

Hypoxic injury in premature infants affects millions of infants and leads to long-term disabilities including problems with motor function. That hypoxia disrupts locomotor function has been known, but the mechanisms mediating hypoxia’s effects have yet to be elucidated. Here we investigate the role of descending projections from the dopaminergic diencephalospinal tract (DDT), a group of highly conserved neurons, which in zebrafish are necessary for normal swimming behavior. We found that the DDT projects to spinal motor neurons, and that hypoxia leads to reduced swimming behavior and a concomitant decrease in DDT to motor neuron synapses. These results offer new insight into the evolutionarily conserved neuronal operators critical for locomotor development in vertebrates, and reveal a molecular mechanism of hypoxic injury.

## Introduction

Development of the CNS depends on precise regulation of oxygen levels. Absence of oxygen (anoxia) or low oxygen levels (hypoxia) can cause devastating effects on development, and a concomitant range of molecular, cellular, and neuronal changes (Bonkowsky and Son, 2018). Hypoxia is a major complication associated with premature birth, and can lead to permanent neurologic impairments including attention-deficit hyperactivity disorder, autism, cerebral palsy, motor impairment, epilepsy, or intellectual disability (Bass et al., 2004; Barrett et al., 2007; Saigal and Doyle, 2008; Back, 2014; Salmaso et al., 2014). Each year worldwide an estimated 15 million infants are born prematurely (<37 weeks’ gestation; Di Fiore et al., 2016). While survival rates for premature infants have drastically improved and the total number of ex-premature infants has increased over the past decade (Mathews et al., 2011), therapies to treat neurodevelopmental diseases resulting from prematurity have been lacking (Fanaroff et al., 2007; Hintz et al., 2011).

Accumulating evidence has shown that hypoxia causes altered neuronal connectivity in the brains of children born prematurely (Gozzo et al., 2009; Mullen et al., 2011). However, the mechanisms linking altered neuronal connectivity, defined as axon pathfinding and synaptic connectivity, to changes in motor function or behavior in vertebrates are still poorly understand. Previous work has shown that developmental hypoxia causes axon pathfinding errors in commissural telencephalic neurons, due to activation of the hypoxia-inducible factor (*hif1*) pathway in the developing zebrafish brain (Stevenson et al., 2012; Xing et al., 2015). *In vivo* as well as *in vitro* studies have demonstrated that hypoxia affects synapse development as well (Curristin et al., 2002; Valdez et al., 2007; Milash et al., 2016; Segura et al., 2016; Son et al., 2016). What has been lacking, however, is an understanding of how changes in connectivity in vertebrates alter behavior. In contrast, experiments in the nematode *Caenorhabditis elegans* have shown that altered connectivity after hypoxia causes behavioral changes including altered responses to sensory stimuli (Chang and Bargmann, 2008; Pocock and Hobert, 2010).

The vertebrate motor system offers a well-characterized model for understanding functional effects of hypoxia-associated connectivity changes. Development of motor function is a tightly regulated process, involving genetically encoded programs and specification of neuronal types and connections (Garcia-Campmany et al., 2010), but also feedback from environmental pathways, such as central pattern generators (CPGs; Berg et al., 2018; D'Elia and Dasen, 2018). The neurotransmitter dopamine has been identified as a critical mediator in several distinct aspects of motor development: dopamine is a brain-derived signaling factor affecting neurogenesis in the spine (Popolo et al., 2004; Reimer et al., 2013); descending projections from the dopaminergic diencephalospinal tract (DDT) are required for vertebrate locomotor maturation; and it is required for regulation of locomotion (Jay et al., 2015; Sharples et al., 2015). Further, tyrosine hydroxylase (TH) immunoreactivity, a marker for synthesis of dopamine, has been shown juxtaposed to motor neurons (McLean and Fetcho, 2004a).

To explore hypoxia’s roles on the interrelationship of motor function and circuitry, we investigated changes in the DDT and motor neuron connectivity, and accompanying effects on locomotor development. We developed transgenic animals expressing fluorescently tagged markers in the DDT and motor neurons to probe for colocalization and proximity at synapses. We found that DDT synaptic proteins immediately juxtapose motor neuron synapses. Since previous studies had shown that hypoxia affected DDT synapses (Son et al., 2016), we characterized the effects of developmental hypoxia on the neurons and circuitry of the DDT and motor neurons. We found no change between hypoxic and normoxic conditions in the number of motor and dopaminergic neurons, or in the axon projections of the DDT to the spinal cord. However, in hypoxic conditions there was a decrease in the number of synapses observed between the DDT and motor neurons. The loss of motor neuron synapses corresponded to a decrease in swimming behaviors compared with normoxic conditions. The impairment in swimming behavior persisted into adulthood, suggesting that developmental hypoxic injury leads to permanent changes in circuitry controlling locomotion.

## Materials and Methods

### Ethics statement

All zebrafish experiments were performed in accordance with guidelines from our institute’s. Animal Care and Use Committee, regulated under federal law (the Animal Welfare Act and Public Health Services Regulation Act) by the United States Department of Agriculture (USDA) and the Office of Laboratory Animal Welfare at the National Institutes of Health, and accredited by the Association for Assessment and Accreditation of Laboratory Animal Care (AAALAC) International.

### Fish stocks, animal husbandry, transgenic line generation

Adult fish were bred according to standard methods. Embryos were raised at 28.5°C in E3 embryo medium with methylene blue, and embryos beyond 24 h postfertilization (hpf) were treated with phenylthiourea (PTU) to prevent pigment formation. For *in situ* staining and immunohistochemistry, embryos were fixed in 4% paraformaldehyde (PFA) in PBS overnight (O/N) at 4°C, washed briefly in PBS with 0.1% Tween 20, dehydrated stepwise in methanol (MeOH; 25%, 50%, 75%, 100%), and stored in 100% MeOH at −20°C until use.

Transgenic fish lines and alleles used in this paper were the following: Tg(*otpb.A:Gal4-VP16_413 − 470_; myl7:EGFP))*^zc57^ (referred to as Tg(*optb.A:Gal4*); Stevenson et al., 2012); Tg(*zcUAS:PSD95.FingR-GFP-ZFC(CCR5TC)-KRAB(A))*^zc88^ (Son et al., 2016); Tg(*otpb.A:EGFP-caax*)^zc49^ (Gutnick et al., 2011); and Tg(*myl7:EGFP*; *UAS:TagRFP-caax*)^zc61^ (Xing et al., 2015). The lines Tg(*otpb.A:TRFP-caax*)^zc97^ and Tg(*Hb9:Gal4VP16_413-470_*)^zc98^ (referred to as Tg(*Hb9:Gal4*)) were generated for this project. Cloning for use in zebrafish was based on the Tol2 kit and recombination reactions with Gateway (Invitrogen) plasmids (Kwan et al., 2007). Identity of constructs was confirmed by restriction enzyme digests and by sequencing of both strands (for coding sequences). Injection of DNA constructs and generation of stable transgenic lines was performed using standard protocols (Bonkowsky et al., 2008). Lines are available on request from the Zebrafish International Resource Center (ZIRC, Eugene, OR) or from the authors.

### Hypoxia reagents

For hypoxia experiments, we followed settings and conditions established in previously published protocols (Stevenson et al., 2012; Xing et al., 2015; Gao et al., 2018). In brief, embryonic zebrafish were placed in a sealed Plexiglas chamber connected via a controller that monitored and adjusted nitrogen gas flow to a desired pO_2_ set point (Biospherix Ltd.). We observed that equilibration of oxygen partial pressures in water could take several hours measured with a dissolved oxygen water meter (Control Company). Therefore, we pre-equilibrated all solutions to either normoxia or hypoxia for at least 4 h before use, and transferred embryos into and out of pre-equilibrated solutions. At the desired time, embryos were placed into media that had been equilibrated to the hypoxic conditions. To terminate hypoxia, embryos were returned to media kept in normoxic conditions. Morphologic staging was used to help determine age at fixation for analyses.

### Spontaneous swimming behavior analysis

Larval behavior analysis was performed on 5 d postfertilization (dpf) larvae in 96-well square bottom plates (Krackeler Scientific) using a video analysis software program (Noldus EthoVision). All experiments were performed in temperature-controlled room dedicated for behavior analysis. For spontaneous behavior observation, animals were transferred at 5 dpf to the 96-well plate for 30-min acclimations prior to recording of behavior. The spontaneous behavior was measured for a total of 70 min, including under alternating dark (4 × 10 min) and light (3 × 10 min) conditions and a one-way ANOVA was conducted.

### Immunohistochemistry and double immunohistochemistry

Antibodies used were: rabbit polyclonal anti-TH 1:250 (Millipore AB152), rabbit polyclonal Hb9 1:250 (ThermoFisher) chicken monoclonal anti-GFP 1:250 (Aveslab, GFP1020), rabbit polyclonal anti-phospho-histone H3 (Thr3; EMD Millipore), rabbit anti-TagRFP 1:250 (Evrogen, AB234), Cy-3 anti-rabbit 1:400 (Millipore, AP132C), Alexa Fluor 488 Goat anti-chicken 1:1000 (ThermoFisher, A11039). Double immunohistochemistry for GFP and TH was performed as follows: embryos were fixed in 4% PFA in PBS for 1.5 h at room temperature (RT) and washed briefly in PBS (3 × 5 min) with 0.1% Triton X-100 (PBST). Embryos were then blocked (PBS with 1% BSA, 1% DMSO, 2% goat serum, and 0.1% Triton X-100) for 3 h at RT, and then incubated in blocking buffer with primary antibodies overnight at 4°C. Embryos were washed with PBST, and incubated with secondary antibodies overnight at 4°C.

### Terminal deoxynucleotidyl transferase dUTP nick-end labeling (TUNEL) staining

TUNEL was performed on whole-mount larvae (ApopTag Fluorescein *In Situ* Apoptosis Detection kit; Millipore Bioscience Research Reagents). After standard fixation and dehydration of larvae in 100% MeOH, larvae were rehydrated stepwise into PBS with 0.1% Tween 20 (PBST), permeabilized with 10 mg/ml Proteinase K in PBST at 28°C, washed twice with PBST, refixed for 20 min with 4% PFA, and washed with PBST. Subsequently, 75 μl of equilibration buffer was added to the larvae for 1 h and then removed and replaced with 55 μl of “working-strength” (per Apoptosis Detection kit instructions) terminal deoxynucleotidyl transferase enzyme overnight at 37°C. To avoid drying out the larvae, Eppendorf tubes were sealed with Parafilm. Before use, the anti-digoxigenin conjugate was warmed to RT. The end-labeling reaction was stopped by washing the embryos three times for 15 min each with 2 ml of the stop/wash buffer, followed by three 1-min washes with PBS. Then 65 ml of working-strength sheep anti-digoxigenin rhodamine was added to the embryos overnight at 4°C. Double immunohistochemistry for GFP and TUNEL was performed by TUNEL staining; after washes with PBST, larvae were fixed for 20 min in 4% PFA at RT and washed again with PBST.

### Western blot analysis

Thirty larvae (5 dpf) per group were deyolked by triturating and incubating in deyolking buffer (65 mm NaCl, 1.7 mm KCl, and 1.5 mm NaHCO_3_) for 10 min at RT. Protein was extracted by grinding larval fish with pestle in a 100 μl of lysis buffer (150 mm NaCl, 20 mm Tris-HCl, pH 7.5, 1 mm EDTA, 1% NP-40, and 1% Triton X-100, 1× Halt Protease and Phosphatase Inhibitor Cocktail; Life Technologies) on ice. The extract was centrifuged for 20 min (16,000 × *g* RCF) at 4°C and the supernatant was transferred to a new tube. Total protein concentration was determined by BCA assay (Thermo Scientific Pierce BCA Protein Assay kit, Fisher Scientific). The protein extract was mixed with an equal amount of 2× Laemmli buffer [20% glycerol, 4% SDS, 0.1% bromophenol blue, 0.125 m Tris (pH 6.8), and 2.5% β-mercaptoethanol] and boiled for 3 min. Samples were stored at −20°C until use. A total of 20 μg of total protein was loaded on to each lane of a 4–20% polyacrylamide gel (Mini-Protean TGX Gel Bio-Rad). After electrophoresis proteins were transferred to PVDF membranes and blocked in 3% non-fat dry milk in TBS (50 mm Tris, 0.138 M NaCl, and 2.7 mm KCl; pH 8.0) for 30 min at RT with agitation. Membranes were split, and then incubated in rabbit anti-TH (1:1000) or rabbit anti-β-actin (1:1000) for 2 h at RT, washed in TBS, and then incubated in HRP-anti-rabbit (1:5000 dilution) for 1 h at RT. Following incubation with secondary antibody, membranes were washed extensively in TBS containing 0.05% Tween 20, and then subjected to chemiluminescent detection (Clarity Western ECL Substrate; Bio-Rad). The Western blot analysis, imaging, and quantification were performed with a digital imager (Gel Do XR+System and Image Lab Software, Bio-Rad) in three separate experimental replicates.

### Microscopy, image analysis, and movie analysis

Confocal imaging was performed, and quantification was performed in ImageJ by compiling a sum projection of 75 slices (step size 2.0 μm) into a single z-stack image. For counts at 72 hpf a 340 × 170-pixel box was drawn in the telencephalon at with the ventral edge placed on the dorsal edge of the eye and the rostral edge at the nose; at 30 and 48 hpf, a 450 × 300-pixel box was drawn around the entire brain. Thresholding was applied from a minimum of 125 to a maximum of 255; and the “Analyze Particles” function was used for quantification.

### Statistical analysis

All data are presented as means ± SEM. Analyses comparing dependent measures across hypoxia and normoxia were completed with Student’s *t* test and one-way ANOVA followed by *post hoc* Tukey test. Overall, statistical significance was set at *p* ≤ 0.05.

## Results

### Descending dopaminergic axons form synapses adjoining spinal cord motor neurons

**Figure 1.**
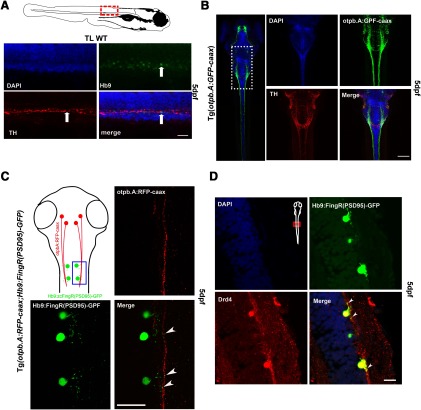
Dopaminergic synapses to spinal cord motor neurons. ***A***, Confocal images, lateral views of zebrafish spinal cord at 5 dpf, dorsal to top; scale bar: 10 µm. DAPI staining of cell nuclei; anti-Hb9 immunohistochemistry in motor neurons; anti-TH immunohistochemistry of DDT; and double-labeling anti-Hb9 anti-TH shows co-localization (arrows). ***B***, Confocal images, dorsal views of zebrafish at 5 dpf, rostral top. Left panel shows entire larva with DDT labeled by GFP in Tg(*otpb.A:EGFP-caax*) line. Higher magnification (scale bar: 50 µm) shows DAPI staining of cell nuclei; GFP labeling of DDT; anti-TH labeling of TH; and co-localization of TH and GFP in DDT. ***C***, Tg(*otpb.A:TRFP-caax*); Tg(*Hb9:FingR(PSD95)-GFP*) larvae, confocal images, dorsal views, of spinal cord at 5 dpf (scale bar: 10 µm). FingR localization of PSD95, expressed in motor neurons, of synapses can be seen neighboring DDT labeled by RFP (arrowheads). ***D***, Anti-D4R labeling co-localizes with FingR GFP expression (scale bar: 10 µm).

We examined the projections of DDT axons, and the proximity of their synapses to spinal cord motor neurons in wild-type zebrafish at 5 dpf. We observed TH-positive (TH^+^) immunostaining juxtaposed with anti-Hb9-positive (Hb^+^) staining of motor neurons in the spinal cord (Fig. 1*A*). To test the source of the TH^+^ immunoreactivity, we co-labeled axons in the Tg(*otpb.A:EGFP-caax*) line, which expresses GFP in the descending DDT (Fujimoto et al., 2011). We found co-expression of TH^+^ and GFP in the DDT (Fig. 1*B*). Next, to evaluate for potential synapse formation between the DDT and motor neurons, we used double transgenic larvae in which a GFP-tagged intrabody (FingR; Gross et al., 2013; Son et al., 2016) labeled the endogenous postsynaptic density 95 (PSD-95) protein at the postsynaptic motor neuron, and RFP-caax labeled the DDT. We found co-localized punctate labeling in the spinal cord of Tg(*otpb.A:TRFP-caax*); Tg(*Hb9:FingR(PSD95)-GFP*) larvae, suggesting the presence of synapses between the DDT and motor neurons (Fig. 1*C*, arrowheads). Interestingly, we identified co-localization of the dopamine receptor 4 (D4R) with the punctate labeling of FingR (PSD95) on motor neurons (Fig. 1*D*). The D4R has been shown to mediate the developmental requirement for dopamine in locomotor maturation and in neurogenesis of motor neurons (Lambert et al., 2012; Reimer et al., 2013). Together, these data suggest the presence of synapses between the DDT and motor neurons in the developing zebrafish spinal cord.

### Hypoxia reduces swimming activity

**Figure 2.**
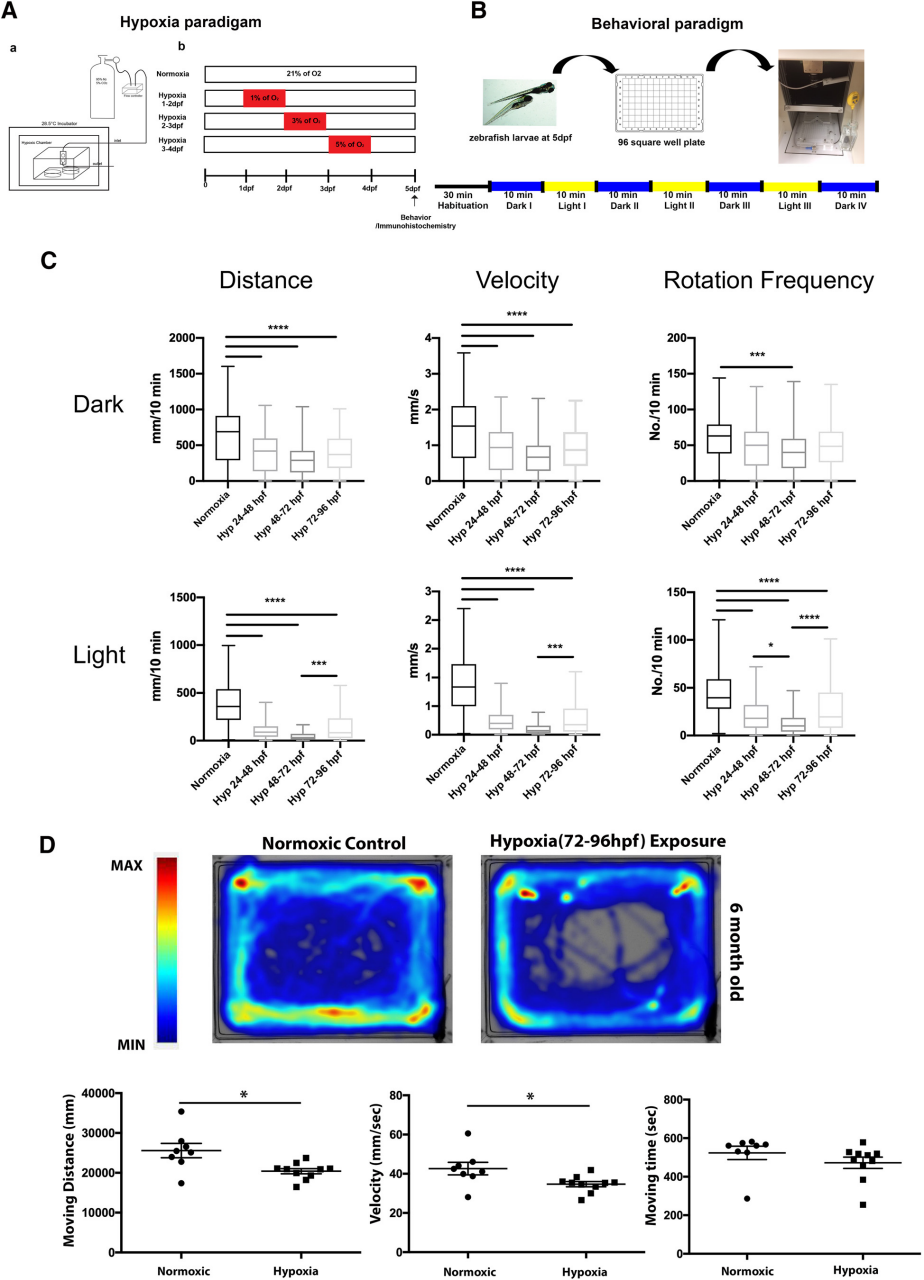
Hypoxia causes impaired motor behavior in larval and adult animals. ***A***, Experimental set-up for hypoxia (***a***) and time line (***b***) of hypoxia exposure and analysis. ***B***, Schematic of behavioral testing paradigm. ***C***, Data plots show impaired motor behavior in hypoxia-exposed larvae, each point is a separate animal. Mean shown, ANOVA, ****p < 0.0001, ***p < 0.001. ***D***, top, Heat-map of adult zebrafish (six-month-old) exposed to hypoxia (3- to 4-dpf 5% pO_2_) shows decreased swimming. Below, Hypoxia-exposed zebrafish have persistent decreases in velocity and distance swum as adults animals (normoxia *n* = 8, pre-hypoxia *n* = 10); *p < 0.05; SD shown).

The DDT is required for locomotor development, and because of our observation of potential synapses between the DDT and motor neurons, we were interested in whether disruption of DDT connectivity would affect motor development. In particular, since hypoxia in developing zebrafish results in pathfinding errors (Stevenson et al., 2012; Xing et al., 2015), and hypoxia has been associated with long-term motor impairment in human infants born prematurely (Martin et al., 2011b; Salmaso et al., 2014), we were interested in whether hypoxia would affect DDT connectivity and lead to resultant swimming deficits. We exposed developing zebrafish larvae to different sub-lethal hypoxic conditions including 1% pO_2_ from 1 to 2 dpf, 3% pO_2_ from 2 to 3 dpf, and 5% pO_2_ from 3 to 4 dpf. The timing and percentage hypoxia was based on previous work, in which these conditions were shown to be non-lethal, and to be non-disruptive to overall development including aspects of CNS development such as cell fate specification and progenitor specification (Stevenson et al., 2012; Xing et al., 2015). Following hypoxia, animals were returned to normoxia, and behavior was then examined at 5 dpf (as the developmental switch in the episodic locomotor (swimming) pattern occurs around 5 dpf; Fig. 2*A*; Buss and Drapeau, 2001; Lambert et al., 2012). Spontaneous swimming behavior was measured for 70 min, including under alternating dark (4 × 10 min) and light (3 × 10 min) conditions (Fig. 2*B*). A one-way ANOVA showed effects of hypoxia on motor behavior, including swimming distance in dark (*F*_(3,111)_ = 11.930, *p* < 0.01) and in light (*F*_(3,111)_ = 14.069, *p* < 0.01); rotation frequency in dark (*F*_(3,111)_ = 2.860, *p* < 0.05) and in light (*F*_(3,111)_ = 6.989, *p* < 0.01); and velocity in dark (*F*_(3,111)_ = 12.175, *p* < 0.01) and in light (*F*_(3,111)_ = 13.793, *p* < 0.01). *Post hoc* comparison using the Tukey HSD test indicated that spontaneous swimming behavior of larval zebrafish exposed to hypoxia was significantly decreased compared with that of larval zebrafish exposed to normoxia (21% pO_2_; Fig. 2*C*; Table 1). Taken together, these results suggest that hypoxia decreases the spontaneous swimming behavior of larval zebrafish and impacts motor development.

**Table 1:** Spontaneous swimming behavior of larval zebrafish at 5 dpf, comparing following hypoxia, to normoxia

Treatment	Swimming distance (mm)		Velocity (mm/s)		Rotation frequency (no/10 min)	
	Dark (4×)	Light (3×)	Dark (4×)	Light (3×)	Dark (4×)	Light (3×)
Normoxia, *n* = 30, 21% pO_2_	616.6 ± 48.3	385.3 ± 38.3	1.4 ± 0.1	0.9 ± 0.1	60.4 ± 3.2	43.2 ± 3.4
Hypoxia 24–48 hpf, *n* = 30, 1% pO_2_	399.5 ± 36.0	197.2 ± 33.2	0.9 ± 0.1	0.4 ± 0.1	50.8 ± 4.6	31.6 ± 5.1
Hypoxia 48–72 hpf, *n* = 25, 3% pO_2_	312.5 ± 28.8	102.9 ± 18.8	0.7 ± 0.1	0.2 ± 0.1	43.3 ± 4.4	18.3 ± 2.6
Hypoxia 72–96 hpf, *n* = 30, 5% pO_2_	391.0 ± 31.6	190.7 ± 28.7	0.9 ± 0.1	0.4 ± 0.1	50.5 ± 34.1	27.9 ± 3.3

Behavioral paradigm used alternating light and dark exposures (see Materials and Methods for full description).

Importantly, we also found that the motor impairments caused by hypoxia persisted into adulthood. In adult fish that had been exposed to hypoxia as 3- to 4-dpf larvae, we also found significant decreases in distance swam (*t*_(16)_ = 2.9, *p* < 0.05) and velocity (*t*_(16)_ = 2.5, *p* < 0.05; but not in total time spent swimming (*t*_(16)_ = 1.1, *p* = 0.27; Fig. 2*D*; Table 2). Together, these results are consistent with hypoxia causing deficits in motor behavior that are sustained through development into adulthood. Further, this suggests that specific changes in motor circuitry may be responsible for the impairment in behavior.

**Table 2:** Motor behavior in adult fish that had been exposed to hypoxia as 3- to 4-dpf larvae, compared with normoxic exposure controls

Treatment	Swimming distance (mm)	Velocity (mm/s)	Movement duration (s)
Control, *n* = 8	25,575.7 ± 1793.7	42.6 ± 3.2	523.4 ± 34.6
Pre-hypoxia 72–96 hpf, *n* = 10, 5% pO_2_	20,285.1 ± 685.6	34.4 ± 1.4	454.1 ± 29.5

### Effects of hypoxia on the development of dopaminergic neurons, DDT, and motor neurons

**Figure 3.**
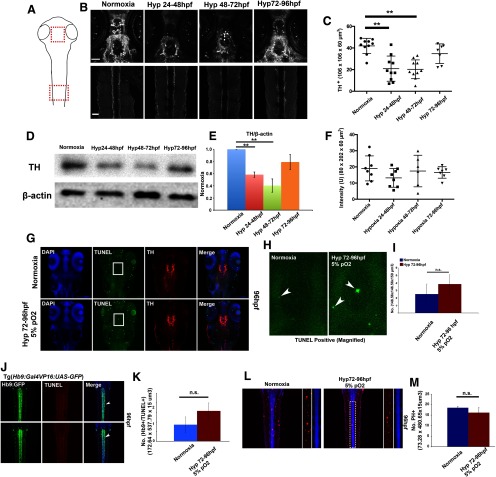
Hypoxia does not alter dopamine or motor neuron numbers, or axon pathfinding of the DDT after hypoxia from 3 to 4 dpf. ***A***, Schematic drawing of regions imaged. Top, Diencephalon (for TH+ neurons). Bottom, Spinal cord (for DDT axons). ***B***, Confocal images, z-stacks, maximum intensity projections, ventral views, rostral to the top; scale bars: 50 µm, anti-TH immunohistochemistry. ***C***, Quantification of number of TH+ neurons in the diencephalon. ***D***, Western blot analysis of whole zebrafish for anti-TH. ***E***, Anti-TH quantification with SD; **p < 0.01. ***F***, Intensity of DDT in the spinal cord shows no decrease following hypoxia. ***G***, ***H***, No increase in apoptosis posthypoxia in the diencephalon. Confocal images, z-stacks, maximum intensity projections, ventral views, rostral to the top, TUNEL labeling in green. ***I***, Quantification with SD of apoptosis following hypoxia. ***J*–*M***, No increase in apoptosis or proliferation posthypoxia in the spinal cord. Motor neurons identified by co-labeling with Tg(*Hb9:GFP*) line. Confocal images, z-stacks, maximum intensity projections, dorsal views, rostral to the top, TUNEL or PH3 (phosphohistone-3) labeling in red. Quantification with SD. n.s., not significant.

To determine where in the CNS hypoxia was exerting its effects, we labeled hypoxic-treated or normoxic-treated 5 dpf zebrafish with anti-TH (Fig. 3*C*,*B*). A one-way ANOVA showed there was a significant effect of hypoxia on the number of TH^+^ neurons in the posterior tuberculum (PT; *F*_(3,33)_ = 13, *p* < 0.01) with certain hypoxia conditions. The *post hoc* analysis revealed there was a significantly reduced number of TH^+^ dopaminergic neurons observed with 1- to 2-dpf hypoxia (1% pO_2_) and 2- to 3-dpf hypoxia (3% pO_2_), but not from 3- to 4-dpf hypoxia (5% pO_2_; Fig. 3*C*). This was also reflected in whole zebrafish anti-TH quantification by Western blot analysis at 5 dpf following hypoxia, in which larval zebrafish showed significantly decreased TH levels at earlier stages of hypoxia exposure including 1–2 dpf (1% pO_2_) and 2–3 dpf (3% pO_2_), but not at 3–4 dpf (5% pO_2_) compared with normoxic zebrafish (*p* = 0.91; Fig. 3*D*,*E*). We did not see any errors in pathfinding of the DDT of extraneous midline crossing (Fig. 3*B*, bottom row); and overall intensity of the DDT descending tracts was unchanged following hypoxia (Fig. 3*F*).

To determine whether hypoxia could specifically alter CNS circuitry, in the absence of changes in apoptosis or cell fate that could cause secondary changes (e.g., following hypoxia at 1–2 or 2–3 dpf), we focused analysis of hypoxia at later developmental stages (3–4 dpf with 5% pO_2_). That is, we did not want to have confounding effects introduced by the more severe defects caused by earlier hypoxia, such as the reduction in TH+ neurons or altered axons (Fig. 3*B*, top panels). We counted the number of TH^+^ neurons in the PT of the diencephalon at 5 dpf and did not find significant changes in counts following hypoxia exposure (normoxia, 41.9 ± 2.2; hypoxia, 34.7 ± 3.4, *t*_(15)_ = 1.848, *p* = 0.08; Fig. 3*C*). Nor were there any difference in apoptosis (measured by TUNEL+ counts) in hypoxia-treated animals (*t*_(12)_ = −1.213, *p* = 0.249; Fig. 3*G–I*). We also evaluated axon pathfinding of the longitudinal DDT track, using the transgenic reporter line Tg(*otpb.A:egfp-caax*) or with anti-TH immunohistochemistry. There were no observed axon pathfinding errors (Fig. 3*B*,*G*), and no change in the intensity of anti-TH labeling [normoxia, 19.09 average intensity units (AI) ± 2.73; hypoxia, 16.5 ± 1.34 AI, *t*_(13)_ = 0.795, *p* = 0.44; Fig. 3*E*,*F*], which is suggestive that the axons are not changing routes. We also characterized the effects of hypoxia on motor neurons in the spinal cord. There were no significant hypoxic effects on proliferation counts (*t*_(10)_ = 0.898, *p* = 0.390) or apoptosis counts (*t*_(12)_ = −1.213, *p* = 0.249) in motor neurons (Fig. 3*J–M*). Together, these data suggest that 5% pO_2_ hypoxia from 3 to 4 dpf did not affect dopaminergic or motor neuron cell counts, apoptosis, or proliferation; and did not affect the axonal projections of the DDT.

### Effects of hypoxia on the synaptic connectivity of DDT to MN

**Figure 4.**
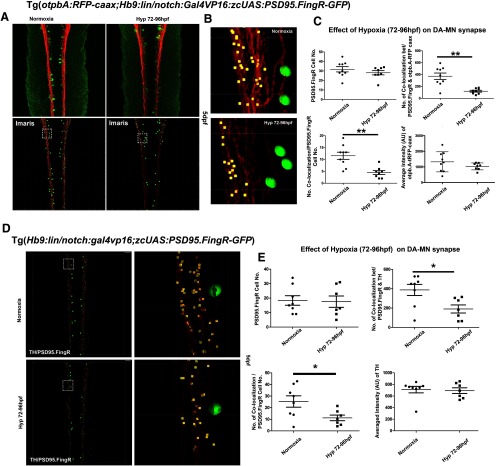
Hypoxia causes a decrease in DDT/motor neuron synapses. ***A***, Confocal images of spinal cord of Tg(*otpb.A.:TRFP-caax*); Tg(*Hb9:FingR(PSD95)-GFP*) larvae, anti-GFP, anti-RFP labeling. Rostral to top, DDT labeled in red, motor neurons in green, synapses in yellow. ***B***, Magnified view of IMARIS imaging of synapses. ***C***, Quantification, mean and SD, **p < 0.01. Number of motor neurons and intensity of DDT is not affected by hypoxia, but number of synapses is decreased, including when corrected for motor neuron number. ***D***, Confocal images of spinal cord of Tg(*Hb9:FingR(PSD95)-GFP*) larvae with anti-TH anti-GFP immunohistochemistry. Right panels, IMARIS panel image shown to right of corresponding confocal image. ***E***, Quantification with mean and SD shows that number of motor neurons and intensity of DDT is not affected by hypoxia, but number of synapses is decreased, including when corrected for motor neuron number. *p < 0.05.

We quantified the synapses between the DDT and motor neurons following hypoxia, using quantification of FingR+/TH+ puncta following anti-TH immunohistochemistry; or of FingR+/RFP+ puncta in double transgenic larvae Tg(*otpb.A:TRFP-caax*); Tg(*Hb9:FingR(PSD95)-GFP*). FingR(PSD95)-GFP is an antibody-like protein that label endogenous synaptic PSD-95 with a GFP label (Son et al., 2016). We analyzed the number of motor neurons expressing FingR(PSD95)-GFP (i.e., number of PSD95-GFP+ motor neurons); the co-localized punctate labeling between FingR(PSD95)-GFP and the DDT (i.e., number of putative synapses); and DDT signal intensity (i.e., axonal projections; Fig. 4*A–E*). In the double transgenic animals, we did not find differences in the number of FingR(PSD95)+ motor neurons (*t*_(15)_ = 1.014, *p* = 0.327), or in the DDT signal intensity (*t*_(15)_ = 1.219, *p* = 0.229; Fig. 4*C*). However, we found a significant decreased number of the co-localized punctate number of FingR(PSD95)-GFP onto DDT in hypoxic zebrafish (120.25 ± 16.10) compared with normoxic zebrafish (372.66 ± 53.72; *t*_(15)_ = 4.267, *p* < 0.01; Fig. 4*C*). Further, when we corrected synapse counts for the number of motor neurons, our results also showed a significant decreased of the number of synapses between the DDT and motor neurons (*t*_(15)_ = 3.955, *p* < 0.05). We observed similar results for quantification using anti-TH immunohistochemistry in Tg(*Hb9:FingR(PSD95)-GFP*) larvae (*t*_(13)_ = 2.471, *p* < 0.05; Fig. 4*D*,*E*). Together, these data indicate that there was a significant decrease in DDT to motor neuron synapses following hypoxia.

**Figure 5.**
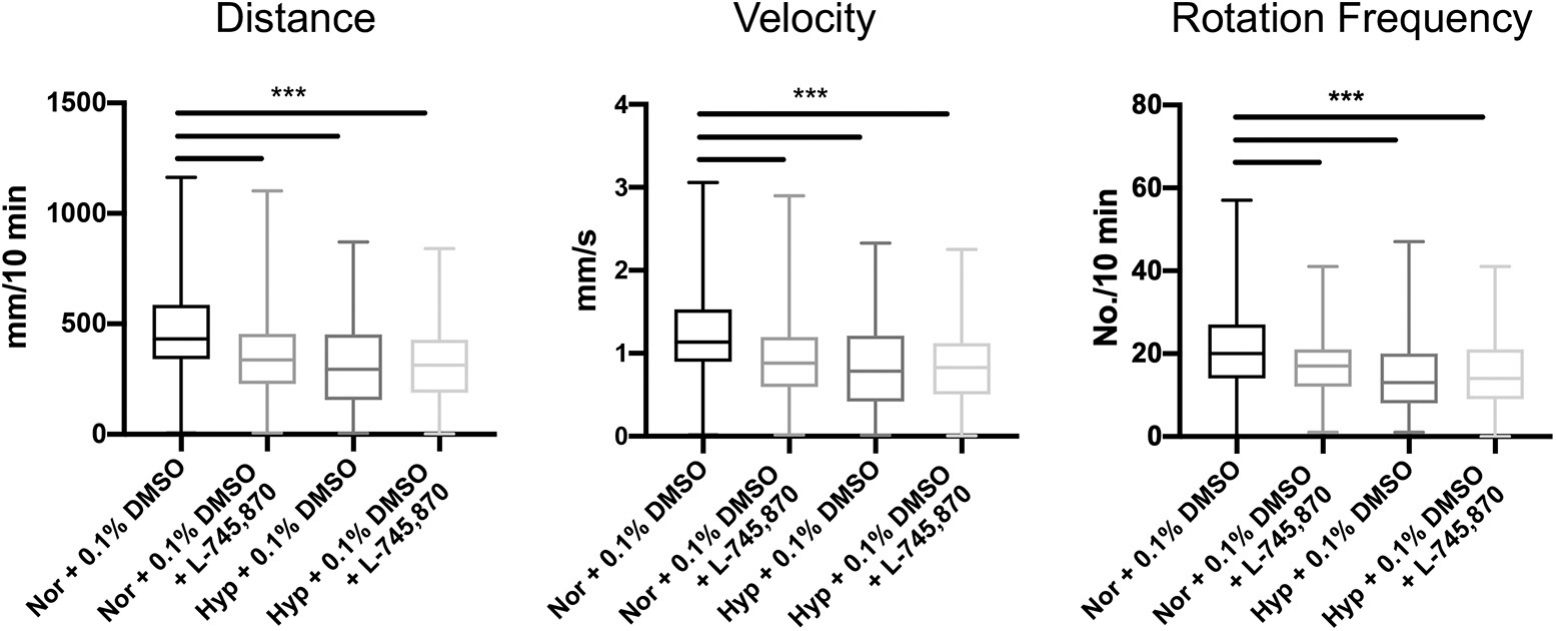
Dopaminergic antagonist exposure impairs zebrafish locomotor development similar to effects of hypoxia. Analysis of zebrafish larvae at 5 dpf, following exposure to L-745870 from 72 to 96 hpf. Impairments in distance, velocity, and rotation frequency (quantified data in Table 3). Each point is a separate animal. Mean shown, ANOVA; ***p < 0.001. Hyp, hypoxia; Nor, normoxia.

Similarly, when exposed zebrafish larvae to a dopaminergic D4 receptor antagonist (L-745870) from 72 to 96 hpf, we observed motor impairments in distance, velocity, and rotation frequency [distance (*t*_(58)_ = 3.90, *p* < 0.01), velocity (*t*_(58)_ = 3.19, *p* < 0.01), and rotation frequency (*t*_(58)_ = 3.93, *p* < 0.01); Fig. 5; Table 3], similar to the effects of hypoxia exposure. Further, inclusion of the D4 receptor antagonist together with hypoxia exposure did not cause any additional worsening of motor phenotypes, consistent with the two exposures acting via a shared or similar mechanism.

**Table 3:** Motor behavior in zebrafish larvae following hypoxia or normoxia, with or without exposure to a dopaminergic D4 receptor antagonist (L-745870) from 72 to 96 hpf

Treatment	Swimming distance (mm)	Velocity (mm/s)	Rotation frequency (no/10 min)
Normoxia, *n* = 24, 21% pO_2_	655.4 ± 71.3	1.7 ± 0.2	54.0 ± 4.4
Hypoxia 72–96 hpf, *n* = 24, 5% pO_2_	361.7 ± 54.1	0.9 ± 0.1	41.5 ± 5.3
D4 control (0.1% DMSO), *n* = 12	581.7 ± 113.5	1.3 ± 0.3	52.1 ± 8.8
D4 antagonist (L-745870), *n* = 12	282.5 ± 37.4	0.6 ± 0.1	23.4 ± 3.7

## Discussion

Our work demonstrates that dopaminergic signaling is necessary for the development of the locomotor synaptic circuitry, and that disruption of the synaptic circuitry impairs motor behavior development. Hypoxia disrupts normal co-regulation of locomotion and synaptogenesis, altering CNS circuitry and leading to persistent deficits in motor behavior. These findings refine our understanding of the role for dopamine, which had previously been demonstrated to be necessary for neurogenesis in the spinal cord, and for proper locomotor development (Popolo et al., 2004; Reimer et al., 2013). Although projections of the A11 group of diencephalic dopaminergic neurons into the spinal cord were first characterized in the 1970s (Björklund and Skagerberg, 1979; Hökfelt et al., 1979), early work on the DDT suggested that the DDT terminated in the dorsal horn and was primarily involved in autonomic functions (Skagerberg et al., 1982). However, TH immunoreactivity was identified near motor neurons in the spinal cord (McLean and Fetcho, 2004b), and functional and behavioral studies revealed the DDT is the source of dopamine involved in motor system development (Lambert et al., 2012), and is necessary for on-going regulation of locomotion behavior (Jay et al., 2015; Sharples et al., 2015). Since we and others have now shown roles for dopamine in motor circuitry development and in motor behavior development, this raises the possibility for a coordinating role for dopamine signaling in these processes.

We found new evidence regarding the role of dopamine in co-regulation of circuitry development with locomotor development. We used the zebrafish model system, characterizing the motor neuron synapses of the spinal cord and the descending dopamine projections to them. We found a decrease in DDT to motor neuron synapses, which was associated with a decrease in swimming behaviors compared with normoxic conditions. Previous work in vertebrates as well as in invertebrates has shown that developmental hypoxia causes axonal pathfinding errors in telencephalic neurons, through activation of the hypoxia-inducible factor (*hif1*) pathway (Stevenson et al., 2012; Xing et al., 2015). Hypoxia has also been noted to affect synapse development (Curristin et al., 2002; Segura et al., 2016; Zhuravin et al., 2019), but in this work we identified that hypoxia could act on dopaminergic signaling to affect synapse development, including effects on synaptic circuitry, and short- and long-term behavioral effects. However, we cannot exclude that hypoxia might have multifactorial impacts on behavior and locomotor development, for example, that hypoxia could be affecting myelin development or function; or of other aspects of circuitry, for example, motor neuron to muscle synapses.

This work also found new evidence for co-localization of DDT descending tract synapses with motor neuron synapses. This finding, if also observed in humans, could provide novel insights into unexplored aspects of human locomotion as well as into pathologies associated with premature birth. In mammals the A11 DDT is also dopaminergic (Koblinger et al., 2014). Premature birth is associated with hypoxic exposures during a critical period of brain development (Martin et al., 2011b, 2012) from 24 to 36 weeks PCA (postconception age), and includes when axon and synaptic connections are forming. This period includes corticospinal tract extension and innervation of spinal primary motor neurons; development of the corpus callosum; thalamocortical axon formation; and the connection of cortical with subcortical areas (Eyre et al., 2000; ten Donkelaar et al., 2004; Haynes et al., 2005; Ren et al., 2006; Kostovic and Jovanov-Milosevic, 2006; Vasung et al., 2010). Hypoxia from prematurity is strongly correlated with the risk for neuromotor impairment (Martin et al., 2011b; Salmaso et al., 2014) and can affect up to 60% of infants (Williams et al., 2010). The hypoxia is secondary to a complex set of conditions, including utero-placental insufficiency and immaturity of the cardiopulmonary system. Following birth, premature infants can experience up to 600 hypoxic episodes per week, each lasting at least >10 s (Martin et al., 2011b).

The impaired locomotion from the developmental hypoxia persists into adulthood. The findings may offer insight into the adverse motor effects of prematurity and accompanying hypoxia. Each year, 13 million infants worldwide are born prematurely (Beck et al., 2010; Martin et al., 2011a), and motor impairment affects a majority of children born prematurely (Williams et al., 2010) with little insight into pathophysiology and no available treatments. Key future research areas include understanding the interaction between myelination and oligodendrocytes, the effects on circuitry from hypoxia, and the relationship of hypoxia-related circuitry changes to complex neurobehavioral disorders such as autism (Yuen et al., 2014; Modabbernia et al., 2017; Wang et al., 2018; Yang et al., 2018; Chen et al., 2019).

## Synthesis

Reviewing Editor: Robert Kalb, Northwestern University Decisions are customarily a result of the Reviewing Editor and the peer reviewers coming together and discussing their recommendations until a consensus is reached. When revisions are invited, a fact-based synthesis statement explaining their decision and outlining what is needed to prepare a revision will be listed below.

The authors have generated important and interesting data. They have responded well to the prior critiques

## Author Response

This manuscript shows that the DDT neurons of the zebrafish express dopamine. The authors follow on to show that DDT synaptic development and behaviors mediated by these neurons can be adversely affected by hypoxic exposure. The experiments seem to be well performed with appropriate controls used in general. However, the conclusions drawn are not fully supported by the experimental outcomes. These issues are detailed below along with minor issues in the text and scientific descriptions that will help with the ability of non-specialists to decipher the study.

Major changes:

1. Line 293 - the authors show that a dopaminergic antagonist cause motor impairments and say that they are similar to those observed upon hypoxic exposure. This suggests to the reader that these two perturbations are linked. However, they could have completely independent effects. The authors should perform the D4 antagonist experiment in the context of hypoxia and examine whether the effect is additive or not. This would help to support the conclusion suggested.

Response

We have now performed an experiment using the D4 dopaminergic antagonist (L745,870), and include that in our revision. We found that inclusion of the antagonist with hypoxia did not cause any additional worsening of phenotype, consistent with hypoxia or the D4 antagonist working via the same mechanism (Figure 5, Table 3).

2. Line 300 - “Our work demonstrates that dopaminergic signaling is necessary for the coordinated development of the motor system and of the locomotor circuitry”.

I don't think that this is a valid conclusion based on the results presented. The authors have shown that specific neurons stain for TH (Figure 1) and that a dopaminergic antagonist can cause motor impairment (Figure 5 and Table 3) but these data do not support a coordinated developmental function for dopamine in regulating development of the motor system and of the locomotor circuitry.

Response

We appreciate the reviewer's concerns about the wording in our Discussion. We have revised our text to be more precise about our findings and to address the reviewer's concerns about our interpretation of the results: (“Our work demonstrates that dopaminergic signaling is necessary for the development of the locomotor synaptic circuitry, and that disruption of the synaptic circuitry impairs motor behavior development. ”); and at the end of the first paragraph of the Discussion as well (“Since we and others have now shown roles for dopamine in motor circuitry development and in motor behavior development, this raises the possibility for a coordinating role for dopamine signaling in these processes.”).

3. Line 319 - “in this work we were able to specifically identify the mechanism (dopamine signaling) by which hypoxia acted, the functional changes in circuitry, and the short- and long-term behavioral effects.” The data present do not support this conclusion. The authors do show that TH expression is reduced by hypoxia. However, the mechanistic association between hypoxia-induced changes in TH expression and circuit/behavioral changes was not substantiated by experimental evidence.

Response

The reviewer identifies important aspects requiring improved clarification in the Discussion, which we have corrected and/or modified to more precisely reflect the experimental results, in the revision (“... but in this work we identified that hypoxia could act on dopaminergic signaling to affect synapse development, including effects on synaptic circuitry, and short- and long-term behavioral effects. ”).

4. Minor changes

Line 219 - the reason for studying the effect of hypoxia on DDT function is unclear. A more robust link between the first two sections needs to be communicated.

Response

We apologize that our chain of logic was unclear- we have expanded this section to clarify why we choose this study of topic.

Line 227 - Don't the authors mean to write alternating rather than alternatively?

Response

We have corrected this.

Line 228 - Change to “A one-way ANOVA was conducted”

Response

We have changed this.

Lines 227- 232 - Super long unwieldy sentence needs to be changed.

Response

We have edited it to make it more readable.

Line 237 - change “from hypoxia” to ‘caused by hypoxia’

Response

Edited.

Figure 2D is labelled 6 month. What does this refer to?

Response

We have fixed the figure to read “6 month old”; and which we also indicate in the Figure legend.

Line 255 Where is the evidence for this sentence? “We did not see any errors in pathfinding of the DDT of extraneous midline crossing”

Response

We have edited the text to indicate that this data is provided in Figure 3B, bottom row of panel images.

Line 263 - the authors show data for TUNEL+ counts but do not explain what this shows. For the non-specialist reference must be made to apoptosis.

Response

We have edited this line to indicate that this is a measure of apoptosis.

Line 266 - “There were no observed axon pathfinding errors” Where is the evidence for this?

Response

We have edited this, including which figures are indicated (“There were no observed axon pathfinding errors (Fig. 3B, G), and no change in the intensity of anti-TH labeling (normoxia, 19.09 average intensity units (AI) {plus minus} 2.73; hypoxia, 16.5 AI {plus minus} 1.34, t(13) = 0.795, p = 0.44) (Fig. 3E, F), which is suggestive that the axons are not changing routes.”).

Line 276 - The pO2 used in Figure 3 is not indicated.

Response

We have edited the figure in the relevant portions (panels G - M).

Line 277 - FingR+ needs to be defined the first time it is mentioned in the results section. What precisely does this marker show?

Response

We have edited the text to describe the FingR reagent (“FingR(PSD95)-GFP is an antibody-like protein that label endogenous synaptic postsynaptic density 95 (PSD-95) with a GFP label (Son et al., 2016).”).

Line 293 - the authors mention a dopaminergic antagonist but do not explain the nature of this intervention. I assume it inhibits a receptor but this is not described.

Response

We now indicate that it is a dopamine D4 receptor antagonist.

Would be appropriate to include the first mechanistic study that revealed hypoxia-induced neurodevelopmental defects (Pocock and Hobert, 2008 Nature Neuroscience)?

Response

We thank the reviewer for noticing this oversight, and have edited the text in the Discussion to reference Pocock and Hobert.

Reviewer: 2

The authors, of manuscript “Dopaminergic Co-Regulation of Locomotor Development and Motor Neuron Synaptogenesis is Uncoupled by Hypoxia in Zebrafish”, has used the zebrafish model system characterizing the motor neuron synapses of the spinal cord and the descending dopamine projections to them. Then demonstrate that dopaminergic signaling is required for the coordinated development of the motor system and of the locomotor circuitry. They reported that hypoxia disrupts normal co-regulation of locomotion and synaptogenesis, altering CNS circuitry and leading to persistent deficits in motor behavior. This offers a new insight into the evolutionarily conserved neuronal operators critical for locomotor development in vertebrates, and reveal a cellular mechanism of hypoxic injury. The manuscript, in this current version, has lots of improvement space.

Major comments:

1) Why did not measure the dissolved absolute oxygen concentration in rearing water using your dissolved oxygen water meter (Control Company)? That will directly give the real hypoxia condition to the larva. Pls refer the “Hypoxia induced retinopathy model in adult zebrafish” (Nature protocol).

Response

We did perform these experiments in our set-up of our hypoxia model, but because of the need to maintain blinding of the reviewer to our identity, we could not at the time of submission list this in our methods/references.

In addition, we have now repeated and performed direct measurements of the hypoxia with a dissolved oxygen meter. Specifically, we found that atmospheric hypoxia correlated directly with larval dissolved water oxygen measures.

2. The choose of different pO2 used in different age of zebrafish larva, and the comparison of results among these groups were not discussed.

Response

We now clarify in more detail why we used the pO2 targets, and the choice of hypoxia timing (“The timing and percentage hypoxia was based on previous work, in which these conditions were shown to be non-lethal, and to be non-disruptive to overall development including aspects of CNS development such as cell fate specification and progenitor specification (Stevenson et al., 2012; Xing et al., 2015).”).

For Figure 3 and subsequent experiments in the paper, in brief, hypoxia at 24-48 and 48-72 had very disruptive effects on the TH neurons and axon pathways, and this would make it extremely difficult to determine what effects of hypoxia and dopamine signaling were due to specific defects caused by hypoxia or altered dopamine signaling; and which effects were non-specific, for example, because of not all the axons reaching their targets, or a decrease in the number of TH+ neurons.

To make this less confusing, we have clarified this in the text (“To determine whether hypoxia could specifically alter CNS circuitry, in the absence of changes in apoptosis or cell fate that could cause secondary changes (e.g. following hypoxia at 1-2 or 2-3 dpf), we focused analysis of hypoxia at later developmental stages (3-4 dpf with 5% pO2). That is, we did not want to have confounding effects introduced by the more severe defects caused by earlier hypoxia, such as the reduction in TH+ neurons or altered axons (see in Fig. 3B, top panels). ”).

3. In the figure 3g-m, only the images of 72-96 hpf group were shown, which is not significant difference and may move to supplementary. While the 24-48 48-72 hpf groups were not given.

Response

We apologize that we were unclear about why we used the 72-96 hpf group for the experiments in Panels G - M. In brief, hypoxia at 24-48 and 48-72 had very disruptive effects on the TH neurons and axon pathways, and this would make it extremely difficult to determine what effects of hypoxia and dopamine signaling were due to specific defects caused by hypoxia or altered dopamine signaling; and which effects were non-specific, for example, because of not all the axons reaching their targets, or a decrease in the number of TH+ neurons.

To make this less confusing, we have clarified this in the text (“To determine whether hypoxia could specifically alter CNS circuitry, in the absence of changes in apoptosis or cell fate that could cause secondary changes (e.g. following hypoxia at 1-2 or 2-3 dpf), we focused analysis of hypoxia at later developmental stages (3-4 dpf with 5% pO2). That is, we did not want to have confounding effects introduced by the more severe defects caused by earlier hypoxia, such as the reduction in TH+ neurons or altered axons (see in Fig. 3B, top panels). ”).

4. It is suggested to provide a rescue experiment after hypoxia to see if activate dopaminergic neurons could partially rescue the locomotion at least.

Response

This is an interesting idea, and we tested it by performing the hypoxia experiment, in the setting of a genetic background of a double-transgenic for UAS:ChR2 (channelrhodopsin2), driven by Gal4 in the dopaminergic neurons (otpb.A:Gal4). So the final experimental set-up was normoxia versus hypoxia, +/- 470nm blue-light exposure, of UAS:ChR2; otpb.A:Gal4 fish.

We found that this approach does not rescue the locomotor behavior:

In retrospect, this result is not so surprising, although it is an appealing idea. In fact, we had used this approach in a previous paper (ChR2 activation). However, in this previous paper, we had shown that the phenotype we measured was activity-dependent, and that loss of activity (as from hypoxia), could be rescued by artificial restoration of activity with ChR2. In this current manuscript, in contrast, we found that hypoxia causes a loss of synapses, and the effect of hypoxia is not mediated by neuronal activity leading to a loss of synapses.

5. The spontaneous swimming behavior test suggests that hypoxia decreases motor development. Certainly the specific changes in motor circuitry might be the major role accounting for the impairment in behavior, while it could not be absolutely exclude other possibilities including motor neuron-muscle and/or axonal myelin impairment. These could be discussed more.

Response

The reviewer makes an excellent point, and in the Discussion we acknowledge these limitations and points raised (“However, we can not exclude that hypoxia might have multifactorial impacts on behavior and locomotor development, for example, that hypoxia could be affecting myelin development or function; or of other aspects of circuitry, for example, motor neuron to muscle synapses.”).

Minor points:

1. In the Fig1A, the white arrows should be noted in legend;

2) Figure3d western gel, four lines of actin intensity varies remarkably;

3)Figure3e Y axis should be normalized of Normoxia either omit the % , or Y axis as 20\40\60\80\100.

4) All the figure letters should unified as A,B,C or a,b,c (Figure 1 and 2 show A,B,C while Figure 3 shows a,b,c).

Response

We have made all of these corrections (please note: the actin intensity varies because of differences in the protein preparation; Figure 3e is normalized to normoxia- the % was incorrectly labeled).

2. How many experiments of the counts of DA-MN synapse have been conducted? Seems the counts data are somewhat scattering.

Response

We have repeated the experiment three times. The scatter appears to be related to fish-fish variability.

3. Figure 5, the * in the Rotation Frequency is not accurately placed.

Response

We have fixed this.

4. Line 10-14, “we found a decrease in the number of synapses from the descending dopaminergic diencephalic tract (DDT) to spinal cord motor neurons”. “Should add in “under hypoxia” condition.

Response

We have edited this.

5. Line 45, “disease” should be “diseases”.

Response

We have fixed this.

6. Line 69, “To explore the interrelationship of functional and circuitry effects of hypoxia,” improper phrase.

Response

We have edited this phrase to make it clear.

7. Line 103, “was” should be replaced “were”.

Response

Thanks- fixed.

8. Line 111, “For hypoxia experiments, we used previously published protocols were followed”. improper phrase.

Response

We have edited this to make it clearer.

9. Fig1B, no scale bar in the left vertical view of larvae, otpb.A:GPF-caax should be otpb.A:GFP-caax.

Response

The scale of this image is the same as the other images.

10. Fig2A, 37{degree sign}C incubator? 28{degree sign}C is suitable for zebrafish.

Response

Thank you for catching that mis-labeling.

11. Fig3b no scale bar.

Response

We thank the reviewer and have corrected this- the scale bars were deleted in the submission process.

12. Fig4, legend is not matched to figure only A and B in the legend. No C-E.

Response

We have corrected the figure legend.
